# Supplementary material for: Impact of clinical pathways on enhancing compliance with evidence-based therapies for Heart failure with reduced ejection fraction–A retrospective cohort study
Source: PLoS One. 2025 Sep 4;20(9):e0330188. doi: 10.1371/journal.pone.0330188 (PMC12410806; doi:10.1371/journal.pone.0330188)
Supplement: S1 File — (PDF) [file pone.0330188.s001.pdf]

Why should I use this pathway?

- **Rapid diuretic titration improves outcomes and decreases LOS** (click to jump to rapid diuretic titration section)
- **Guideline-directed medical therapy (GDMT) decreases readmission and mortality** (click to jump to GDMT section)
- **Sudden cardiac death (SCD) assessment decreases readmission and mortality** (click to jump to SCD assessment)

Daily checklist

1. Titrate diuretics
2. Titrate GDMT to goal
3. Complete Sudden Cardiac Death Assessment prior to discharge
4. Identify and resolve **barriers to discharge**

Pathway context

- Authors / references / metrics
- Patient education videos

Adult inpatient with heart failure with left ventricular ejection fraction ≤ 40% (HFrEF)

↓

Evidence of Congestion?

Clinical indicators & triggers for volume overload

NO → **Ensure patient is on appropriate GDMT**

Click to link to GDMT pathway

YES ↓

Complete initial evaluation, monitoring, and education orders

- **Assess** risk factors, family history, symptoms, and exam findings
- Click here to see most recent weights

Order initial evaluation, monitoring, and education orders:

- **Labs:** BNP / HS Troponin / AM LFTs / HbA1c / lipid panel / TSH / AM CBC / AM BMP
- **Monitoring:** telemetry / I/Os / daily weights
- **Education:** nursing heart failure education
- **Nurse Navigation:** Consult to HF Nurse Navigator

Monitoring serial BNP is not helpful in assessing response to diuresis, check at admission and at euvolemia / discharge

- ****Click here for additional general admission orders****

Respiratory orders:

- Oxygen via nasal cannula if saturation < 90% OR PaO2 <60 mmHg

Imaging orders (if not already completed):

- EKG / CXR / Echo

Patient education:

Assess for Home Hospital eligibility (currently BH and YNHH only)

Click to order Home Hospital Evaluation consult

- Home Hospital eligibility criteria
- Services provided by Home Hospital
- Benefits of Home Hospital

feedback

Order initial evaluation, monitoring, and education orders:

- **Labs:** BNP / HS Troponin / AM LFTs / HbA1c / lipid panel / TSH / AM CBC / AM BMP
- **Monitoring:** telemetry / I/Os / daily weights
- **Education:** nursing heart failure education
- **Nurse Navigation:** Consult to HF Nurse Navigator

Monitoring serial BNP is not helpful in assessing response to diuresis, check at admission and at euvolemia / discharge

- **Click here for additional general admission orders**

Respiratory orders:

- Oxygen via nasal cannula if saturation < 90% OR PaO2 <60 mmHg

Imaging orders (if not already completed):

- EKG / CXR / Echo

Patient education:

- Click here to access and send Emmi videos directly to your patient's MyChart
- Consult nutrition for dietary education if concerns for poor understanding or compliance with diet

Assess for Home Hospital eligibility (currently BH and YNHH only)

Click to order Home Hospital Evaluation consult

- Home Hospital eligibility criteria
- Services provided by Home Hospital
- Benefits of Home Hospital

Place consult orders, if indicated

- **For all cases of newly documented LVEF ≤ 40%, order:**
  - YNHH Cardiology consult
  - BH/GH/LMW Cardiology consult
- **YNHH:** consult Heart Failure team if patient meets criteria
- **For ALL heart failure patients not already being followed, order:**
  - Referral to comprehensive heart failure management (to facilitate long-term disease management)

Complete daily checklist

1. **Titrate diuretics** using Diuretic Titration Pathway
2. **Titrate GDMT to goal** using GDMT Pathway
3. **Complete sudden cardiac death assessment before discharge** using Sudden Cardiac Death Assessment Pathway
4. **Identify and resolve** barriers to discharge

These recommendations reflect YNHHS consensus based on review of existing evidence and guidelines. They do not replace clinical judgement

feedback

Why should I use this Pathway?

- Rapid diuretic titration improves outcomes and decreases LOS
- Rapid diuretic titration involves reassessment 4-6 hours after initiation/dose change and subsequent dose escalation if inadequate response as outlined on this pathway

Daily checklist

1. Titrate diuretics
2. Titrate GDMT to goal
3. Complete Sudden Cardiac Death Assessment prior to discharge
4. Identify and resolve barriers to discharge

Adult inpatient with heart failure who is congested based on history, exam, or labs/imaging

- Clinical indicators and triggers for volume overload
- Click here to see most recent weights

Diuresis best practices

- **Every 4 hour assesment** for first 24 hours until response
- Once daily dosing is rarely effective
- Diurese until at dry weight or set new dry weight after decongested
- Re-assess dosing / eGFR / potassium with addition of SGLT2 or aldactone
- Monitoring serial pBNP is not helpful in assessing response to diuresis. Check at admission and at euvolemia / discharge

Acute decline in eGFR or eGFR < 20 mL/min?

YES → **Review medication modifications and recommended consults**

If chronic eGFR < 20, doubling pathway diuretic recommendations may improve diuretic response

- Indications to hold RAAS blockade
- Click for consult recommendations and orders

NO → **On diuretics prior to admission?**

NO → **Start initial diuretics (diuretic-naive patient)**

Diuretic orders (choose one):

Furosemide	Bumetanide

YES → **Initiate 2.5x diuretic dosing algorithm**

Consult Cardiology if algorithm suggests ≥ 160 mg IV lasix or ≥ 8 mg IV Bumex. Suggested doses can be altered at the discretion of the prescribing provider. **Dosing recommendations are in the absence of metolazone. If patient is on metolazone at baseline, consult cardiology for loop diuretic dosing suggestions**

Diuretic orders (choose one):

feedback

Start initial diuretics (diuretic-naive patient)

Diuretic orders (choose one):

Furosemide	Bumetanide
I/Os Q4h / Furosemide 40 mg IV Once	I/Os Q4h / Bumetanide 1 mg IV Once

Potassium repletion orders:

- Nurse driven protocol where available
- Oral potassium repletion
- IV potassium repletion

Initiate 2.5x diuretic dosing algorithm

Consult Cardiology if algorithm suggests ≥ 160 mg IV lasix or ≥ 8 mg IV Bumex. Suggested doses can be altered at the discretion of the prescribing provider. **Dosing recommendations are in the absence of metolazone. If patient is on metolazone at baseline, consult cardiology for loop diuretic dosing suggestions**

Diuretic orders (choose one):

Furosemide	Bumetanide
I/Os Q4h / Furosemide IV once	I/Os Q4h / bumetanide IV once

Dosing considerations (example):

- If furosemide, multiply individual (40 mg daily or BID would yield the same inpatient dose) outpatient dose by 2.5 and give as IV bolus q 12 hours
- If bumetanide multiply individual outpatient dose by 2.5 and give as IV bolus q 12 hours
- Use same medication IV as is used outpatient
- Dosing Table
  - Torsemide conversion to bumetanide or furosemide, then dose as above
- Click for potassium repletion orders

Re-Evaluate in 4 hours

Responding 4 hours post diuretic?
Daily diuresis goals

- Responding defined as > 750mL total urine output at 4h
- If unable to obtain accurate urine output, can check spot urine sodium 1-2 hours after loop diuretic. If spot urine sodium is < 60 mmol/L- patient is not responding
- Assess for improvement in symptoms and exam

Not responding / **Responding**

Maximize loop diuretic therapy and reassess

1. Maximize diuretic: double furosemide or bumetanide dose IV 4 hours after initial dose

- Furosemide IV BID OR
- Bumetanide IV BID

2. Reassess: 4 hours after administration

- **Responding defined as > 750mL total urine output at 4h**
- Assess for improvement in symptoms / exam

Responding

Not responding

Continue bumetanide IV BID **OR** furosemide IV BID **at effective dose**

Daily evaluation

Complete daily evaluation **for decongestion**

feedback

Maximize loop diuretic therapy and reassess

1. **Maximize diuretic:** double furosemide or bumetanide dose IV 4 hours after initial dose
   - Furosemide IV BID OR
   - Bumetanide IV BID
2. **Reassess:** 4 hours after administration
   - **Responding defined as > 750mL total urine output at 4h**
   - Assess for improvement in symptoms / exam

Responding → Continue bumetanide IV BID OR furosemide IV BID at effective dose

Not responding ↓

Titrate diuretics and review criteria for cardiology consultation
(if not already consulted)

Diuretic titration and reassessments:
- Continue to double diuretic dosing and reassess every 4 hours for response until reaching dose of furosemide 160 mg IV BID or bumetanide 8 mg IV BID
- Assess barriers to effective diuresis
  - Unable to quantify output or weight
  - Declining renal function
  - Urinary retention

Indications for cardiology consult:
- Not responding to diuresis
- Furosemide ≥ 160 mg IV BID OR bumetanide ≥ 8 mg IV BID
  - Cardiology team will guide addition of thiazide diuretic (such as metolazone) and consideration of transfer to higher level of care
  - **Orders:**
    - YNHH Cardiology consult
    - BH/GH/LMW Cardiology consult

↓

Continue to assess for inadequate response/worsening hemodynamics

- Worsening electrolyte abnormalities - hyponatremia
- Evidence of organ malperfusion - rising creatinine, change in mental status

If not responding, next steps will be considered by Cardiology team:
- CHF team evaluation
- Right heart catheterization

Continue bumetanide IV BID **OR** furosemide IV BID **at effective dose**

Daily evaluation ↓

Complete daily evaluation for decongestion

- Indications that decongestion has been achieved

Renal considerations:
- If not fully decongested, do not interupt diuresis provided creatinine increase ≤ 0.5
- If sodium falling < 130-132, consider free water restriction < 1.5 liters

NOT decongested → Continue bumetanide IV BID OR furosemide IV BID at effective dose

Decongested ↓

Transition to oral diuretics

Important considerations at euvolemia:
- Evaluate need for ongoing diuretic therapy after decongestion
- Goal to transition to oral form of inpatient loop diuretic
- Ensure Guideline Directed Medical Therapy

Diuretic dosing guidance:
- **For patients on diuretics prior to admission:**
  - Outpatient diuretic dosing for patients on diuretics prior to admission
- **For patients newly initated on diuretics:**
  - Outpatient diuretic guidance if receiving furosemide ≤ 40 mg IV or bumetanide ≤ 1 mg IV BID
  - Outpatient diuretic guidance if receiving furosemide > 40 mg IV or bumetanide > 1 mg IV BID

Orders:
- Furosemide PO or bumetanide PO or torsemide PO
  - Loop diuretic conversion chart
- BNP (check at euvolemia)

feedback

- Unable to quantify output or weight
- Declining renal function
- Urinary retention

Indications for cardiology consult:

- Not responding to diuresis
- Furosemide ≥ 160 mg IV BID OR bumetanide ≥ 8 mg IV BID
  - Cardiology team will guide addition of thiazide diuretic (such as metolazone) and consideration of transfer to higher level of care
  - **Orders:**
    - YNHH Cardiology consult
    - BH/GH/LMW Cardiology consult

Continue to assess for inadequate response/worsening hemodynamics

- Worsening electrolyte abnormalities - hyponatremia
- Evidence of organ malperfusion - rising creatinine, change in mental status

If not responding, next steps will be considered by Cardiology team:

- CHF team evaluation
- Right heart catheterization
- Review Echo
- Consideration of increased level of care

If responding to diuretics: click to jump to "responding to diuretics guidance"

Decongested

Transition to oral diuretics

Important considerations at euvolemia:

- Evaluate need for ongoing diuretic therapy after decongestion
- Goal to transition to oral form of inpatient loop diuretic
- Ensure Guideline Directed Medical Therapy

Diuretic dosing guidance:

- **For patients on diuretics prior to admission:**
  - Outpatient diuretic dosing for patients on diuretics prior to admission
- **For patients newly initated on diuretics:**
  - Outpatient diuretic guidance if receiving furosemide ≤ 40 mg IV or bumetanide ≤ 1 mg IV BID
  - Outpatient diuretic guidance if receiving furosemide > 40 mg IV or bumetanide > 1 mg IV BID

Orders:

- Furosemide PO or bumetanide PO or torsemide PO
  - Loop diuretic conversion chart
- BNP (check at euvolemia)

Coordinate ambulatory diuretic monitoring

- Evaluation of response to oral diuretics may take place at initial follow up visit (< 7 days) and/or after 12-24 hours post transition to oral diuretics
- Schedule follow up in 5-7 days at disease management clinic/cardiology
- Order BMP to be drawn prior to outpatient follow-up, cc results to outpatient cardiologist
  - **Order:** outpatient BMP
- Educate about weight gain post discharge
- Educate about appropriate urinary reponse to diuretic therapy
- Educate about potential side effects/symptoms of overdiuresis

These recommendations reflect YNHHS consensus based on review of existing evidence and guidelines. They do not replace clinical judgement

feedback

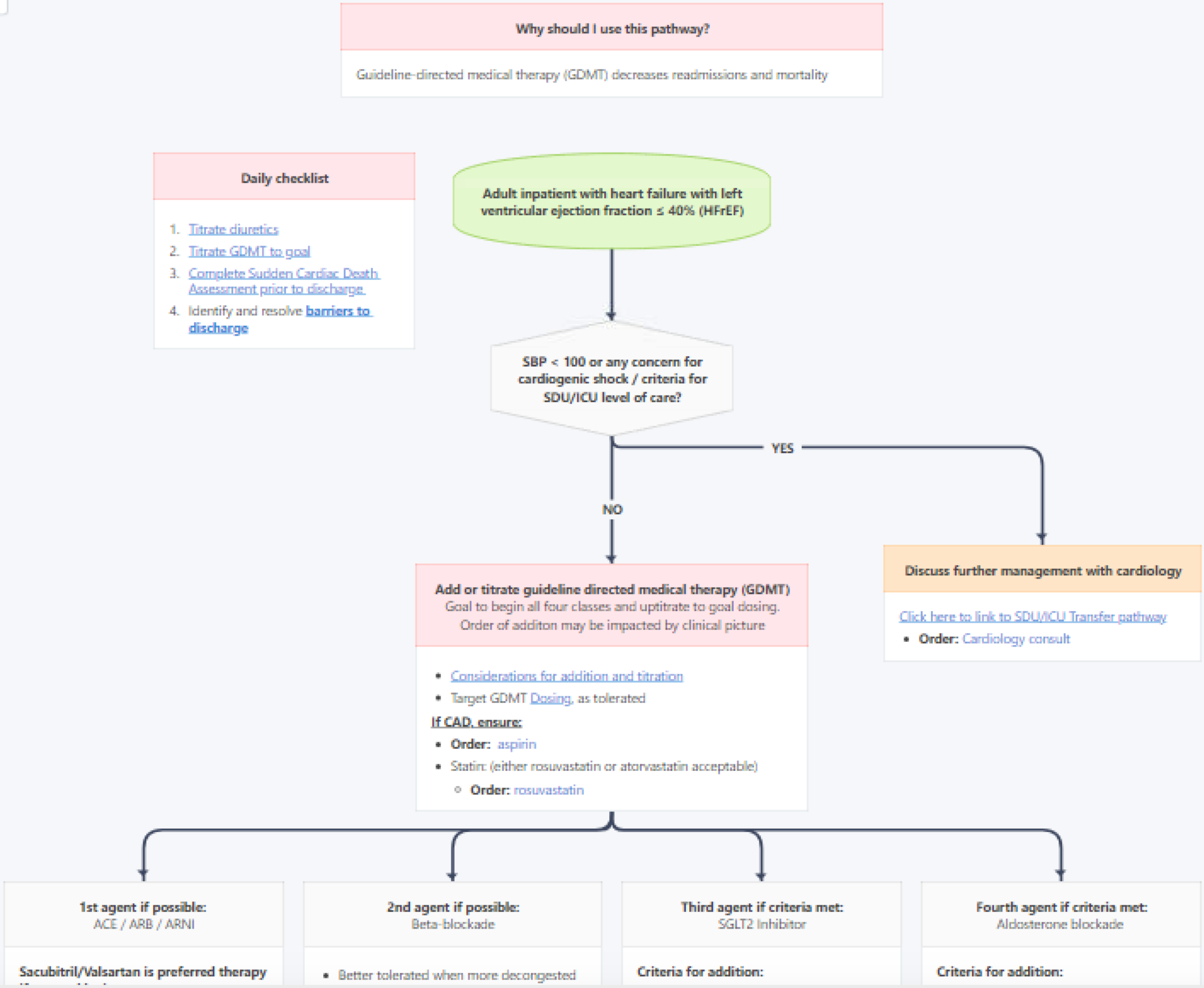
Why should I use this pathway?
Guideline-directed medical therapy (GDMT) decreases readmissions and mortality
Daily checklist
1. Titrate diuretics
2. Titrate GDMT to goal
3. Complete Sudden Cardiac Death Assessment prior to discharge
4. Identify and resolve barriers to discharge
Adult inpatient with heart failure with left ventricular ejection fraction ≤ 40% (HFrEF)
SBP < 100 or any concern for cardiogenic shock / criteria for SDU/ICU level of care?
YES
NO
Add or titrate guideline directed medical therapy (GDMT)
Goal to begin all four classes and uptitrate to goal dosing.
Order of additon may be impacted by clinical picture
• Considerations for addition and titration
• Target GDMT Dosing, as tolerated
If CAD, ensure:
• Order: aspirin
• Statin: (either rosuvastatin or atorvastatin acceptable)
○ Order: rosuvastatin
Discuss further management with cardiology
Click here to link to SDU/ICU Transfer pathway
• Order: Cardiology consult
1st agent if possible:
ACE / ARB / ARNI
Sacubitril/Valsartan is preferred therapy
2nd agent if possible:
Beta-blockade
• Better tolerated when more decongested
Third agent if criteria met:
SGLT2 Inhibitor
Criteria for addition:
Fourth agent if criteria met:
Aldosterone blockade
Criteria for addition:

feedback

- Target GDMT dosing, as tolerated

If CAD, ensure:

- **Order:** aspirin
- Statin: (either rosuvastatin or atorvastatin acceptable)
  - **Order:** rosuvastatin

1st agent if possible:
ACE / ARB / ARNI

Sacubitril/Valsartan is preferred therapy if covered by insurance

- Check to see if patients meet criteria for treatment initiation
  - **Order:** sacubitril / valsartan 24/26 mg BID OR increase to next dose
  - Up-titration is reasonable during hospitalization pending BP and renal function

Caution use in patients with:

- Recent increase in IV diuretics
- Recent use of IV vasodilators or inotropes
- Consider use of ARB in patient in patients with higher risk of hypotension

If sacubutril / valsartan not covered, add ACE inhibitor or angiotensin II receptor blocker

If patient on GDMT, but not on Entresto, may be candidate for HIC 200028712

Study Title: Mechanisms underlying hypotensive response to ARB/NEP Inhibition
https://clinicaltrials.gov/ct2/show/NCT04649229
Contacts: Lindsey.Douglas@yale.edu
Sub-Investigator:
Christopher.Maulion@yale.edu
Phone: 203-583-1874
Principal Investgator: Nancy J. Brown, MD

2nd agent if possible:
Beta-blockade

- Better tolerated when more decongested
- If significant bradycardia, discuss with Cardiology prior to initiation
- If not on beta-blocker at admission, initiate evidence based low dose beta-blockade:
  - **Preferred therapy (order):** carvedilol BID
  - If tachyarrhythmia or history of tachyarrythmia: metoprolol succinate may be preferred (not metoprolol tartrate)
- If already on beta blocker: **uptitrate as tolerated**
  - **Goal:** Carvedilol 25 mg BID **OR**
  - **Goal:** Metoprolol succinate 200 mg QD

Heart rate above goal (>70bpm) in sinus ryhthm despite maximum tolerated beta blockade **and** euvolumic **and** compensated?

Ivabradine - should only be added by cardiology or advanced CHF team. check for cost as well

Third agent if criteria met:
SGLT2 Inhibitor

Criteria for addition:

- GFR > 25
- Confirm outpatient insurance coverage
  - If not covered, empagliflozin may be an alternative as an outpatient
- Not approved for patients with type I Diabetes

Order: Dapagliflozin

- Some patients may need dose reduction of loop diuretic at discharge. Monitor renal function and diuretic response
- Need repeat BMP in 1 week after initiation
- If > 30% decrease in GFR, consider discontinuation, renal input

Fourth agent if criteria met:
Aldosterone blockade

Criteria for addition:

- On ACE/ARB/ARNI
- Cr ≤ 2.5 men, ≤ 2 women , GFR ≥ 30
- K ≤ 5
- If K > 5, consider K+ binders - SZC, Patiromer
- If history of non-compliance, consider holding until follow up
- Must be able to comply with blood work every 3 months

Order:

- Spironolactone 12.5 to 25mg daily
- BMP - within 72 hours of start

If a male patient has a history of gynecomastia with spironolactone, please call pharmacy for review of eplerenone alternative agent

These recommendations reflect YNHHS consensus based on review of existing evidence and guidelines. They do not replace clinical judgement

feedback

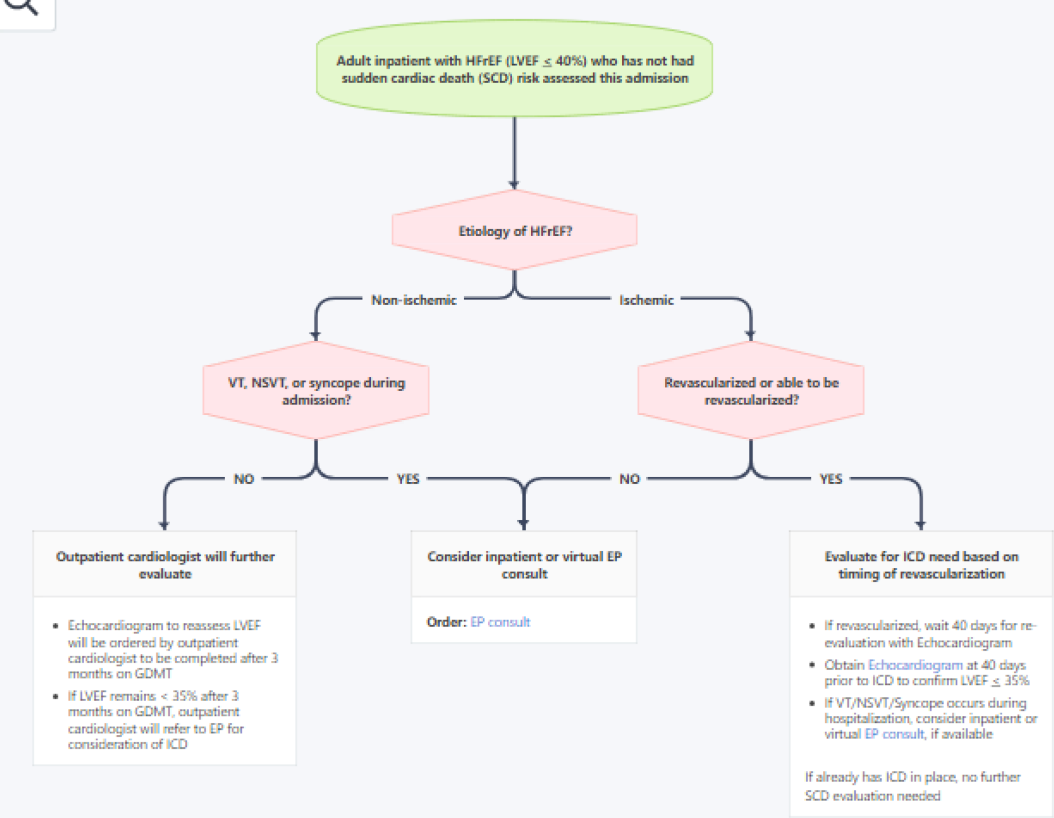
Adult inpatient with HFrEF (LVEF ≤ 40%) who has not had sudden cardiac death (SCD) risk assessed this admission
Etiology of HFrEF?
Non-ischemic
Ischemic
VT, NSVT, or syncope during admission?
Revascularized or able to be revascularized?
NO
YES
NO
YES
Outpatient cardiologist will further evaluate
Echocardiogram to reassess LVEF will be ordered by outpatient cardiologist to be completed after 3 months on GDMT
If LVEF remains < 35% after 3 months on GDMT, outpatient cardiologist will refer to EP for consideration of ICD
Consider inpatient or virtual EP consult
Order: EP consult
Evaluate for ICD need based on timing of revascularization
If revascularized, wait 40 days for re-evaluation with Echocardiogram
Obtain Echocardiogram at 40 days prior to ICD to confirm LVEF ≤ 35%
If VT/NSVT/Syncope occurs during hospitalization, consider inpatient or virtual EP consult, if available
If already has ICD in place, no further SCD evaluation needed

feedback

Adult inpatient with heart failure with evidence of acute decompensation

- Congestion **or**
- Worsening hemodynamics **or**
- Declining functional status

Trigger identification best practices

- **Multiple triggers may be present**
- Identify and address barriers to compliance
- Provide appropriate education and social work assistance
- Document triggers for prognosis and risk of readmission
- Place appropriate referral to cardiology and cardiology subspecialties

What are the suspected decompensation triggers?

Acute injury	Arrhythmia	New or worsening valvular disease	Other
• Ischemia<br>• Aortic dissection<br>• Pulmonary embolism	• New or rapid a-fib/a-flutter<br>• Awake heart rate < 50<br>• Type II second degree AV block (Mobitz 2)<br>• Complete heart block	• Severe acute MR/TR<br>• Severe AS, AR, or MS<br>• Mild/moderate valvular disease	• Click to view other potential etiologies

Other →

Exclude additional potential triggers (multiple triggers may be present)

- Medication or dietary adherance **(diagnosis of exclusion)**
- Recent medication changes
- Underlying medical illness
- Uncontrolled hypertension
- Worsening renal failure or cardio-renal syndrome
- New or worsening anemia
- Worsening cardiomyopathy
- RV pacing / wide QRS
- Alcohol or substance abuse
- Endocrinologic disorders

Acute injury →

Rule out ischemia, aortic dissection, or pulmonary embolism

Ischemia:

- Revascularization if indicated per cardiology
- Ensure optimal Guideline Directed Medical Therapy (GDMT) for coronary artery disease

Concern for aortic dissection:

- **Order:** CTA Chest

Concern for PE:

- Click to go to Embolism Pathway

Arrhythmia →

Manage new or worsening arrhythmias/conduction system disease

Type of arrhythmia or valvular disease	Next steps	Appropriate orders

Valvular disease →

Determine next steps based on valve condition

Condition	Next steps

feedback

• Aortic dissection<br>• Pulmonary embolism	< 50<br>• Type II second degree AV block (Mobitz 2)<br>• Complete heart block	• Severe AS, AR, or MS<br>• Mild/moderate valvular disease	• Click to view other potential etiologies

Acute injury

Arrhythmia

Valvular disease

Rule out ischemia, aortic dissection, or pulmonary embolism

Ischemia:

- Revascularization if indicated per cardiology
- Ensure optimal Guideline Directed Medical Therapy (GDMT) for coronary artery disease

Concern for aortic dissection:

- **Order:** CTA Chest

Concern for PE:

- Click to go to Embolism Pathway

Exclude additional potential triggers (multiple triggers may be present)

- Medication or dietary adherence **(diagnosis of exclusion)**
- Recent medication changes
- Underlying medical illness
- Uncontrolled hypertension
- Worsening renal failure or cardio-renal syndrome
- New or worsening anemia
- Worsening cardiomyopathy
- RV pacing / wide QRS
- Alcohol or substance abuse
- Endocrinologic disorders

Manage new or worsening arrhythmias/conduction system disease

Type of arrhythmia or valvular disease	Next steps	Appropriate orders
New or rapid a-fib or a-flutter	**Optimize rate control and anticoagulation:**<br>• Beta-blockade preferred after stabilization of decompensated CHF<br>• Non-dihydropyridine CCB's are contraindicated<br>• Anticoagulation for all patients suggested regardless of CHADSVasc2 if no contraindication<br>• Rhythm control recommended for most patients ultimately	**If resting average heart rate > 100 bpm and ongoing clinical decompensation or ongoing atrial flutter:**<br>• Inpatient consult to Cardiology (or EP) (YSC/SRC) (BH/GH/LMW) for consideration of anti-arrhythmic strategy/ablation vs DCCV with appropriate AC strategy<br>**For all other patients:**<br>• Refer for outpatient EP evaluation
• Awake heart rate < 50<br>• Type II second degree AV block (Mobitz 2)<br>• Complete heart block	Reduction in AV nodal blocking agent or pacemaker may be needed	• **Order:** consult to cardiology or EP (YNHH) (BH/GH/LMW)<br>• **Order:** consult to EP (YSC/SRC) (BH/GH/LMW)

Determine next steps based on valve condition

Condition	Next steps
Severe acute MR/TR due to endocarditis or flail leaflet	**Order:** urgent consult to cardiology (YNHH) (BH/GH/LMW)<br>Urgent consult to CT Surgery (YNHH) (BH/GH/LMW)
Severe MR/TR NOT due to endocarditis or flail leaflet	1. Re-evaluate with diuresis if congested and normal valve morphology<br>2. Consult cardiology if no improvement<br>◦ **Order:** consult to cardiology (YNHH) (BH/GH/LMW)
Severe AS, AR, or MS	• **YNHH order:** at discretion of cardiology consult team consult to valve team<br>• **BH/GH/LMH/WH order:** consult to cardiology
Mild/moderate valvular disease	Follow-up at discretion of cardiology

feedback

Pathways

BACK

Heart Failure with Reduced Ejection Fraction (HFrEF): Adult Inpatient

In Progress

Initial Eval / Admission | Titrate Diuretics | Optimize GDMT | Assess SCD Risk | Identify Triggers | Determine Etiology | ICU/SDU Transfer | DC Planning

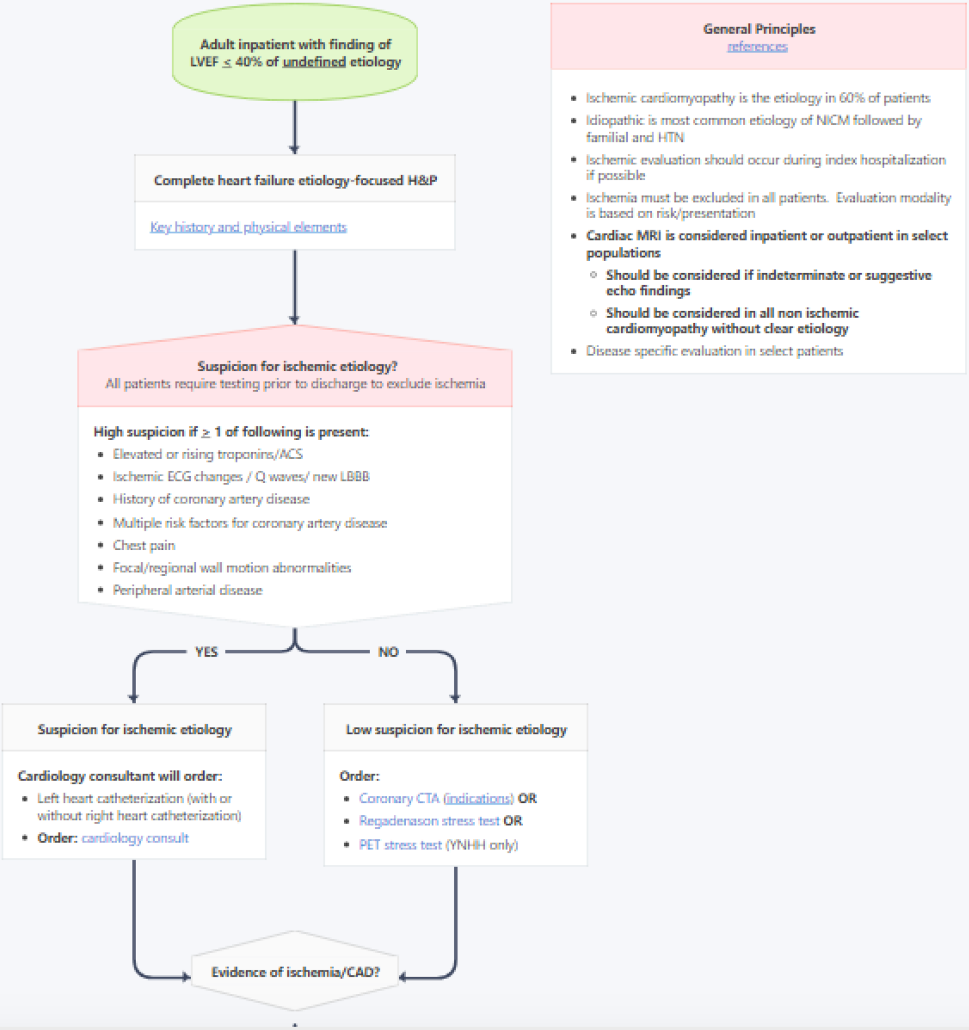

feedback

Pathways

BACK

Heart Failure with Reduced Ejection Fraction (HFrEF): Adult Inpatient

In Progress

Initial Eval / Admission | Titrate Diuretics | Optimize GDMT | Assess SCD Risk | Identify Triggers | **Determine Etiology** | ICU/SDU Transfer | DC Planning

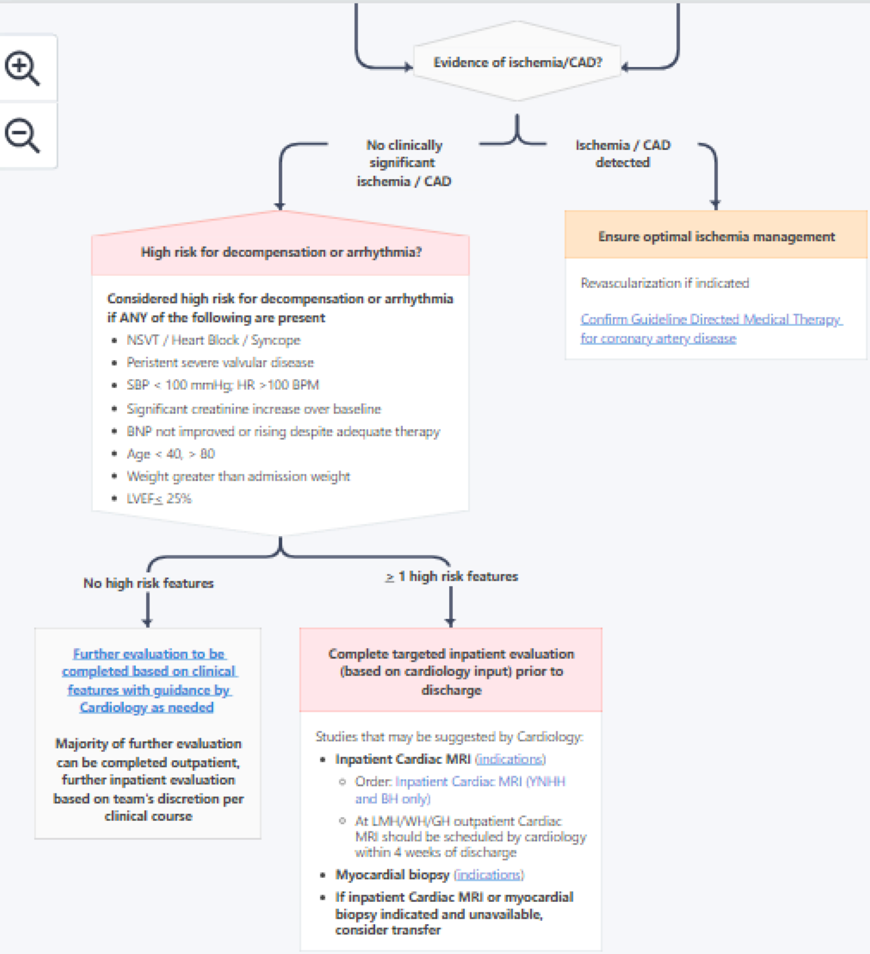

feedback

Meets any of the following CICU / MICU criteria?

**Unstable physiologic parameters**	Click to view unstable physiologic parameters
**Borderline physiologic parameters**	Click to view borderline physiologic parameters
**Uncontrolled arrhythmias**	• Bradyarrhythmias<br>• High-degree AV block
**Cardiogenic shock**	• Hemodynamic clues: persistent hypotension (systolic blood pressure < 90 mm Hg) and reduced cardiac index (< 2.2 L/minute/m2) in presence of elevated pulmonary capillary wedge pressure (such as > 18 mm Hg)<br>• Clinical clues:<br>◦ Low blood pressure<br>◦ Cool extremities-not required<br>◦ Unable to tolerate usual GDMT<br>◦ Mental status changes

YES →

Review Goals of Care

Consider Palliative Care consult if end-stage disease

Consult CICU or MICU for transfer

- **Order:** transfer to ICU
- **Y-access:** ###
- How to transfer patients via Y-access at YNHHS

NO ↓

Meets any of the following SDU / PCU criteria?

**Some unstable physiologic parameters**	• O_2 sat >90% but requiring > 4 L O_2 NC<br>• Borderline HR, RR, BP
**Stable anti-arrhythmic and rate control gtt (varies per DN)**	
**Stable inotropic vasopressor dose for ≥24 hours**	
**Need for high-level / intensive Nursing care**	• Aggressive pulmonary toilet requiring:<br>◦ Suctioning q 2-3 hours nebulizer treatments (continuous or more frequently than every 4 hours)<br>• Need for increased frequency of monitoring<br>◦ Continuous O_2 saturation monitoring<br>◦ Stable Bipap previously initiated in ICU
**At risk for instability**	• Arrhythmias not resulting in severe hemodynamic instability<br>• Initiation of anti-arrhythmic medication<br>• Monitoring requiring arterial catheter (A-Line)<br>• Monitoring requiring central venous pressure (CVP) [illegible]

YES →

Consult SDU or PCU (if available) for transfer

- **Order:** transfer to SDU (PCU)
- [illegible]

feedback

NO

Meets any of the following SDU / PCU criteria?

**Some unstable physiologic parameters**	• O_2 sat >90% but requiring > 4 L O_2 NC<br>• Borderline HR, RR, BP
**Stable anti-arrhythmic and rate control gtt (varies per DN)**	
**Stable inotropic vasopressor dose for ≥24 hours**	
**Need for high-level / intensive Nursing care**	• Aggressive pulmonary toilet requiring:<br>◦ Suctioning q 2-3 hours nebulizer treatments (continuous or more frequently than every 4 hours)<br>• Need for increased frequency of monitoring<br>◦ Continuous O_2 saturation monitoring<br>◦ Stable Bipap previously initiated in ICU
**At risk for instability**	• Arrhythmias not resulting in severe hemodynamic instability<br>• Initiation of anti-arrhythmic medication<br>• Monitoring requiring arterial catheter (A-Line)<br>• Monitoring requiring central venous pressure (CVP) monitoring<br>• Inotropes for HF or vasopressors requiring titration within pharmacy guidelines for step-down bed<br>• Patients requiring increased nursing observation within the limits of step-down care staffing guidelines

YES →

Consult SDU or PCU (if available) for transfer

- **Order:** transfer to SDU (PCU)
- **Y-access:**
- How to transfer patients via Y-access at YNHHS

Can likely remain on the floor unless other non-cardiac indication for transfer to a higher level of care is present

feedback

Refer to Comprehensive Heart Failure Program
(if not already established in program)

- **Order:** Referral to comprehensive heart failure management
  - Available at all locations, write location preference in comments section

All of the following completed?

- Decongestion achieved and transitioned to oral diuretics
- GDMT optimized
- SCD risk assessment completed
- Trigger for decompensation identified and addressed
- Etiology of heart failure defined, if not already established
- Referred to Comprehensive Heart Failure Program (is not already established)
- Barriers to discharge are identified and resolved

NO → **Complete outstanding assessments or further titration of treatment prior to discharge**

YES ↓

Discharge guidance

Click to launch discharge summary

Patient education:

- Click here to send EMMI educational videos to your patient's MyChart
- Click for Epic Clinical References and search for "heart failure" to select educational handouts

Schedule follow-up:

- Cardiology
- PCP
- Referral to comprehensive heart failure management (available at all locations, location preference in comments section)

Ensure patient has a working scale

- If not, order: DME order for scale

Provide patient and family education:

- Click for Epic Clinical References and search for "heart failure" to select educational handouts

feedback
