# Supplementary material for: Impact of clinical pathways on enhancing compliance with evidence-based therapies for Heart failure with reduced ejection fraction–A retrospective cohort study
Source: PLoS One. 2025 Sep 4;20(9):e0330188. doi: 10.1371/journal.pone.0330188 (PMC12410806; doi:10.1371/journal.pone.0330188)
Supplement: S2 File — (DOCX) [file pone.0330188.s002.docx]

| **Supplement 2: List of Heart failure diagnosis codes from ICD-10** | |
| --- | --- |
| I11.0 | Hypertensive heart disease with heart failure |
| I13.0 | Hypertensive heart and chronic kidney disease with heart failure and stage 1 through stage 4 chronic kidney disease, or unspecified chronic kidney disease |
| I13.2 | Hypertensive heart and chronic kidney disease with heart failure and with stage 5 chronic kidney disease, or end stage renal disease |
| I50.1 | Left ventricular failure, unspecified |
| I50.20 | Unspecified systolic (congestive) heart failure |
| I50.21 | Acute systolic (congestive) heart failure |
| I50.22 | Chronic systolic (congestive) heart failure |
| I50.23 | Acute on chronic systolic (congestive) heart failure |
| I50.30 | Unspecified diastolic (congestive) heart failure |
| I50.31 | Acute diastolic (congestive) heart failure |
| I50.32 | Chronic diastolic (congestive) heart failure |
| I50.33 | Acute on chronic diastolic (congestive) heart failure |
| I50.40 | Unspecified combined systolic (congestive) and diastolic (congestive) heart failure |
| I50.41 | Acute combined systolic (congestive) and diastolic (congestive) heart failure |
| I50.42 | Chronic combined systolic (congestive) and diastolic (congestive) heart failure |
| I50.43 | Acute on chronic combined systolic (congestive) and diastolic (congestive) heart failure |
| I50.810 | Right heart failure, unspecified |
| I50.811 | Acute right heart failure |
| I50.812 | Chronic right heart failure |
| I50.813 | Acute on chronic right heart failure |
| I50.814 | Right heart failure due to left heart failure |
| I50.82 | Biventricular heart failure |
| I50.83 | High output heart failure |
| I50.84 | End stage heart failure |
| I50.89 | Other heart failure |
| I50.9 | Heart failure, unspecified |

**Readmission Measures Methodology [Internet]. QualityNet Home; 2024. Available from:* [*https://qualitynet.cms.gov/inpatient/measures/readmission/methodology*](https://qualitynet.cms.gov/inpatient/measures/readmission/methodology)*. Accessed 2024 Aug 2.*
