# Supplementary material for: Impact of clinical pathways on enhancing compliance with evidence-based therapies for Heart failure with reduced ejection fraction–A retrospective cohort study
Source: PLoS One. 2025 Sep 4;20(9):e0330188. doi: 10.1371/journal.pone.0330188 (PMC12410806; doi:10.1371/journal.pone.0330188)
Supplement: Attachment — Submitted filename: Responses to Reviewer comments _F.docx [file pone.0330188.s006.docx]

Dear Editor,

Thank you for sharing the comments from the reviewers, and alerting us to the journal requirements. We appreciate the comments, and we have made updates to the manuscript accordingly. Please find our responses to the comments below.

Sincerely,

Naga Sasidhar Kanaparthy, MD

**Journal Requirements:**

When submitting your revision, we need you to address these additional requirements.

**1. Please ensure that your manuscript meets PLOS ONE's style requirements, including those for file naming. The PLOS ONE style templates can be found at** [**https://journals.plos.org/plosone/s/file**](https://journals.plos.org/plosone/s/file)**id=wjVg/PLOSOne_formatting_sample_main_body.pdf and** [**https://journals.plos.org/plosone/s/file?id=ba62/PLOSOne_formatting_sample_title_authors_affiliations.pdf**](https://journals.plos.org/plosone/s/file?id=ba62/PLOSOne_formatting_sample_title_authors_affiliations.pdf)

We have reviewed PLOS ONE’s style requirements and have made the following updates:

- Title has been changed to sentence case
- Corresponding author name added

**2. Thank you for stating the following in your Competing Interests section:**
**Please complete your Competing Interests on the online submission form to state any Competing Interests. If you have no competing interests, please state "The authors have declared that no competing interests exist.", as detailed online in our guide for authors at** [**http://journals.plos.org/plosone/s/submit-now**](http://journals.plos.org/plosone/s/submit-now)
**This information should be included in your cover letter; we will change the online submission form on your behalf.**

Many thanks for this comment and assistance. None of the authors have any conflicts of interest to report. We have added this line to the cover letter.

**3. Your ethics statement should only appear in the Methods section of your manuscript. If your ethics statement is written in any section besides the Methods, please delete it from any other section.**

The ethics statement now appears only in the Methods section.

**4. We note that you have indicated that there are restrictions to data sharing for this study. For studies involving human research participant data or other sensitive data, we encourage authors to share de-identified or anonymized data. However, when data cannot be publicly shared for ethical reasons, we allow authors to make their data sets available upon request. For information on unacceptable data access restrictions, please see** [**http://journals.plos.org/plosone/s/data-availability#loc-unacceptable-data-access-restrictions**](http://journals.plos.org/plosone/s/data-availability#loc-unacceptable-data-access-restrictions)**.**

Before we proceed with your manuscript, please address the following prompts:

**a) If there are ethical or legal restrictions on sharing a de-identified data set, please explain them in detail (e.g., data contain potentially identifying or sensitive patient information, data are owned by a third-party organization, etc.) and who has imposed them (e.g., a Research Ethics Committee or Institutional Review Board, etc.). Please also provide contact information for a data access committee, ethics committee, or other institutional body to which data requests may be sent.**

N/A

**b) If there are no restrictions, please upload the minimal anonymized data set necessary to replicate your study findings to a stable, public repository and provide us with the relevant URLs, DOIs, or accession numbers. Please see** [**http://www.bmj.com/content/340/bmj.c181.long**](http://www.bmj.com/content/340/bmj.c181.long) **for guidelines on how to de-identify and prepare clinical data for publication. For a list of recommended repositories, please see** [**https://journals.plos.org/plosone/s/recommended-repositories**](https://journals.plos.org/plosone/s/recommended-repositories)**. You also have the option of uploading the data as Supporting Information files, but we would recommend depositing data directly to a data repository if possible.**

**Please update your Data Availability statement in the submission form accordingly.**

The data has since been uploaded to a public data repository : IEEE Dataport

The DOI is : 10.21227/xdqg-6m89

**5. In the online submission form, you indicated that data are available from the Yale Univeristy Institutional Data Access / Ethics Committee for researchers who meet the criteria for access to confidential data.**

**All PLOS journals now require all data underlying the findings described in their manuscript to be freely available to other researchers, either 1. In a public repository, 2. Within the manuscript itself, or 3. Uploaded as supplementary information.**
**This policy applies to all data except where public deposition would breach compliance with the protocol approved by your research ethics board. If your data cannot be made publicly available for ethical or legal reasons (e.g., public availability would compromise patient privacy), please explain your reasons on resubmission and your exemption request will be escalated for approval.**

The data has since been uploaded to a public data repository : IEEE Dataport

The DOI is : 10.21227/xdqg-6m89

**6.** [**Please remove all personal information, ensure that the data shared are in accordance with participant consent, and re-upload a fully anonymized data set.**

**Note: spreadsheet columns with personal information must be removed and not hidden as all hidden columns will appear in the published file.**

**Additional guidance on preparing raw data for publication can be found in our Data Policy (https://journals.plos.org/plosone/s/data-availability#loc-human-research-participant-data-and-other-sensitive-data) and in the following article: http://www.bmj.com/content/340/bmj.c181.long.**](https://nam12.safelinks.protection.outlook.com/?url=http%3A%2F%2Ftrack.editorialmanager.com%2FCL0%2Fhttp%3A%252F%252Fwww.bmj.com%252Fcontent%252F340%252Fbmj.c181.long.%2F1%2F010f01963f817b10-6ccbf5ed-2bff-4fc1-80ed-b7d31645dbc7-000000%2FWb-_pMrWb-m32WH-Jn1a9lEfUmcUsH0YyVTG5zfbank%3D207&data=05%7C02%7Cnaga.kanaparthy%40yale.edu%7Cfb03d5595d3b4b3835f008dd7d06a9e3%7Cdd8cbebb21394df8b4114e3e87abeb5c%7C0%7C0%7C638804190068639034%7CUnknown%7CTWFpbGZsb3d8eyJFbXB0eU1hcGkiOnRydWUsIlYiOiIwLjAuMDAwMCIsIlAiOiJXaW4zMiIsIkFOIjoiTWFpbCIsIldUIjoyfQ%3D%3D%7C0%7C%7C%7C&sdata=b74mlfNw2aZXrAlRTBO91jRNJU%2FlJtthPE4IraoxXjk%3D&reserved=0)

 The data has since been uploaded to a public data repository : IEEE Dataport

**7.** [**Please include captions for your Supporting Information files at the end of your manuscript, and update any in-text citations to match accordingly. Please see our Supporting Information guidelines for more information: http://journals.plos.org/plosone/s/supporting-informatio**n.](https://nam12.safelinks.protection.outlook.com/?url=http%3A%2F%2Ftrack.editorialmanager.com%2FCL0%2Ffile%3A%252F%252F%252FC%3A%252FUsers%252Fe759388%252FDocuments%252F2025%252FMS%2525202025%252FPlease%252520include%252520captions%252520for%252520your%252520Supporting%252520Information%252520files%252520at%252520the%252520end%252520of%252520your%252520manuscript%2C%252520and%252520update%252520any%252520in-text%252520citations%252520to%252520match%252520accordingly.%252520Please%252520see%252520our%252520Supporting%252520Information%252520guidelines%252520for%252520more%252520information%3A%252520http%3A%252Fjournals.plos.org%252Fplosone%252Fs%252Fsupporting-information.%252520%2F1%2F010f01963f817b10-6ccbf5ed-2bff-4fc1-80ed-b7d31645dbc7-000000%2FXI6W-164nacOEAJIRpTFSgQJ6OHI9furweCrTjZWXFE%3D207&data=05%7C02%7Cnaga.kanaparthy%40yale.edu%7Cfb03d5595d3b4b3835f008dd7d06a9e3%7Cdd8cbebb21394df8b4114e3e87abeb5c%7C0%7C0%7C638804190068654339%7CUnknown%7CTWFpbGZsb3d8eyJFbXB0eU1hcGkiOnRydWUsIlYiOiIwLjAuMDAwMCIsIlAiOiJXaW4zMiIsIkFOIjoiTWFpbCIsIldUIjoyfQ%3D%3D%7C0%7C%7C%7C&sdata=33aKUpUQHBWt8OMywxpfmZmiWa38yUXi8qnQbihUviE%3D&reserved=0)

We have clarified Supplement 1 and updated all files to be in compliance with the guidelines.

**REVIWER COMMENTS**

**Reviewer #1: I cannot provide details for the present manuscript if I don't have the tables. I apologize for the incovenience.**

Five tables have been added to the manuscript for your review.

**Reviewer #2: Dear Author,**

**I’ve reviewed the manuscript and would like to prepare a concise and constructive revision report. Here are the suggested changes for improving the manuscript:**

**1. Title and Abstract:**
**The title is appropriate but could be slightly revised to better highlight the significance of care pathways in HFrEF treatment. Consider rephrasing it to something like: "Impact of Clinical Pathways on Enhancing Compliance with Evidence-Based Therapies for HFrEF."**

We appreciate the suggestion and agree with this comment. Title has been updated to:

*“Impact of clinical pathways on enhancing compliance with evidence-based therapies for heart failure with reduced ejection fraction: a retrospective cohort study.”*

**The abstract clearly summarizes the study's purpose, methods, results, and conclusion. However, emphasize the significance of the findings in terms of clinical application and future implications in the conclusion.**

*“Despite low utilization, pathway use was associated with more early diuretics and referrals to CHFM with a trend toward more GDMT on discharge, which is known to enhance survival and quality of life.”*

**2. Introduction:**
**Add a brief sentence explaining why compliance with GDMT is specifically challenging in clinical practice.**
**Mention the gap in literature this study addresses more explicitly to enhance its relevance.**

We have clarified our introduction near line 71 which now reads:

“*GDMT remains challenging in clinical practice due to polypharmacy, patient comorbidities, and care fragmentation. Hospitalization presents a crucial window to initiate comprehensive goal-directed medical therapy (GDMT) with all four agents in heart failure patients, ensuring optimal treatment while under close clinical supervision and addressing the real-world gap of suboptimal GDMT prescription in the ambulatory setting.*”

**3. Methods:**
**Include more detail on how the care pathway was designed and the rationale for selecting its components.**

We have added a sentence beginning on line 122:

*“Its components were derived from existing national guidelines as well as input from front-line clinicians regarding their work-flow and point-of care decision-making.”*

**Provide a brief explanation of the statistical methods used, ensuring clarity for readers without a deep statistical background.**

We have added a sentence beginning on line 170:

*“Logistic regression models estimate the odds of an outcome occurring based on one or more predictor variables.”*

**4. Results:**
**Expand the discussion on the differences observed in early diuretic use and referrals to CHFM in terms of clinical outcomes and patient benefits.**

We have added a paragraph in the Discussion beginning on line 258:

*“Although complete GDMT at discharge did not reach statistical significance—likely reflecting existing prescribing habits and limited integration time—the observed positive trend remains clinically important given GDMT’s known survival benefits. Notably, the significant improvement in ACEI/ARB/ARNI use is particularly impactful due to their well-established benefits on survival and hospitalization rates.”*

**While the use of GDMT was mentioned, elaborate on why it did not reach statistical significance and its implications for the study findings.**

We have added to the Discussion beginning on line 262:

*“The statistically significant increase in early diuretic use is clinically valuable, as timely diuretics improve symptoms and shorten hospital stays. Similarly, increased referrals to comprehensive HF management substantially enhance care continuity, reduce readmissions, and improve patient outcomes.”*

We also deleted a paragraph “*Notably … GDMT.*”

**5. Discussion:**
**Include a brief acknowledgment of potential biases introduced by the voluntary use of pathways.**

To address the issue of bias, we have added specific language both in the Methods and the Limitations section beginning on lines 143 and 316 respectively:

*“While the pathway was equally accessible, its voluntary nature introduces the possibility of self-selection bias, as providers who chose to use the pathway may differ in their clinical practices or engagement with guideline-based care. However, we anticipate minimal bias in outcome measurement, as all relevant data were extracted directly from the EHR using standardized criteria.”*

*“Second, there is potential for selection bias, as physicians who chose to use the pathway may differ systematically from those who did not, possibly due to greater engagement with evidence-based care or differing clinical practices. This self-selection could influence the observed associations between pathway use and improved outcomes.”*

**Highlight the broader implications of implementing similar care pathways in other healthcare settings.**

We have added language in the Discussion beginning on line 301:

*“If adapted and individualized to local clinical workflows and institutional needs, similar care pathways could yield meaningful improvements in guideline adherence and patient outcomes across diverse healthcare settings.”*

**6. Conclusion:**
**Emphasize the study's contribution to the field and outline specific next steps for research or implementation.**

We have made edits to the Conclusion to reflect the study’s contribution to the field that include deleting text and adding new language beginning on line 331:

Deleted lines:

*~~“Care pathways such as the one described in our study provide a vehicle to standardize evidence-based care across a large complex healthcare system and ensure that all providers always have access to the most up to date, expert knowledge available.”~~*

Added lines:

*“This study contributes to the field by demonstrating that EHR-embedded CPs can effectively standardize evidence-based practices within complex healthcare systems. Future research should focus on identifying barriers to broader pathway adoption, evaluating clinical outcomes in diverse settings, and developing tailored implementation strategies to enhance clinician engagement and utilization.”*

**General Improvements:**
**Ensure all abbreviations (e.g., CHFM, GDMT) are clearly defined upon first use.**
**Check for consistency in formatting tables and figures for better readability.**

We reviewed all abbreviations and made the requested edits.

**Reviewer #3: Tables are not seen in the manuscript. Kindly include all the three tables for review.**

The tables have been added.

**The article is well written and stresses the importance of diuretics in early stages of heart failure.**

Thank you for your comment.

**The number of people on extra medications like digoxin can be mentioned in the manuscript.**

We agree that the description about the use of digoxin would have been useful. Unfortunately, these data are not available in our dataset. But we will consider it in our future papers.

**Reviewer #4: Re Manuscript Number PONE-D-24-34453: The authors have presented the results of care pathways for heart failure with reduced ejection fraction within the context of healthcare systems in the United States. The call to action for adhering to guidelines in the hospital setting is admirable. However, the authors should provide more context regarding the low uptake of their intervention in light of more recent evidence (10.1016/j.jacc.2023.12.024). Moreover, elaborating on what distinguishes this study from others in literature (e.g., 10.5837/bjc.2022.005; 10.1002/ehf2.14911) would be beneficial. Further comments are provided below.**

We have added language in multiple locations to address these helpful comments:

Limitations section has been edited beginning on line 311:

*“Our study has several limitations. First, the pathway's low utilization may reflect a common challenge in implementing clinical decision support tools. [26]In our healthcare system, most HF patients are managed by general hospitalists who, due to the routine nature of HF admissions, may not perceive the need to consult a pathway. This perception can lead to underutilization, despite the complexity and evolving nature of HF guidelines, which span over 40 pages.[4] Second, there is potential for selection bias, as physicians who chose to use the pathway may differ systematically from those who did not, possibly due to greater engagement with evidence-based care or differing clinical practices. This self-selection could influence the observed associations between pathway use and improved outcomes.”*

Discussion has been edited beginning on line 297:

*“Building on prior work such as the Program to Optimize Heart Failure Patient Pathways (PRO-HF), which focused on outpatient care, our study expands the evidence base by evaluating an inpatient, EHR-integrated pathway across a large, diverse health system. This distinction underscores the potential for such pathways to standardize care and improve outcomes in the inpatient setting. [25]*

*If adapted and individualized to local clinical workflows and institutional needs, similar care pathways could yield meaningful improvements in guideline adherence and patient outcomes across diverse healthcare settings.”*

Citations have been added:

25.	Patwala, A., et al., *Optimizing heart failure pathways to enhance patient care: the Program to Optimize Heart Failure Patient Pathways (PRO-HF).* ESC Heart Fail, 2024. **11**(5): p. 2578-2590.

26.	Sutton, R.T., et al., *An overview of clinical decision support systems: benefits, risks, and strategies for success.* npj Digital Medicine, 2020. **3**(1): p. 17.

Major comments:

**1) As suggested by PLOS One journal, I would encourage the authors to follow the STROBE reporting guideline in their manuscript (**[**https://www.strobe-statement.org/**](https://www.strobe-statement.org/)**).**

We have submitted a STROBE checklist.

**2) Although not explicitly stated, the theme underlying the arguments presented by the authors in both abstract and introduction sections tends to switch between inpatient and outpatient settings. It is important that authors transparently present arguments relevant only to hospital settings; otherwise, they may risk confusing readers.**

We appreciate this comment and have edited the Introduction beginning on line 71 to emphasize the inpatient nature of our intervention:

*“GDMT remains challenging in clinical practice due to polypharmacy, patient comorbidities, and care fragmentation. Hospitalization presents a crucial window to initiate comprehensive goal-directed medical therapy (GDMT) with all four agents in heart failure patients, ensuring optimal treatment while under close clinical supervision and addressing the real-world gap of suboptimal GDMT prescription in the ambulatory setting.”*

We have also added a clarifying phrase in the Introduction on line 77:

*“Similarly, in the inpatient setting, symptom control with loop diuretics is the mainstay for patients with HF, however early and aggressive diuresis is underachieved [9].”*

**3) Unfortunately, tables were not available for review; however, based on what has been stated in text, I would encourage authors not to compare baseline characteristics of participants via statistical measures as this would not provide clinically meaningful insights. Furthermore, considering a large sample size and assuming a high event rate, I argue that using odds ratios could yield exaggerated results—this is also evident from wide precision margins accompanying effect sizes—a risk difference might provide more meaningful interpretation of findings.**

Thank you for your thoughtful feedback. We have now added the tables to the manuscript for your review. We appreciate your point regarding the interpretation of statistical measures and have clarified the statistical methods accordingly. In consultation with a senior biostatistician at Yale, we have confirmed that multivariable logistic regression was appropriate for our analysis. We have added a brief explanation of this method in the text to aid readers less familiar with statistical modeling. While we acknowledge the limitations of odds ratios, especially in large samples, we believe they still offer value in estimating associations in this context.

**4) Authors tend to dichotomize results based on statistical significance; I discourage this approach and invite interpreting results based on clinical significance instead.**

We have added/edited the Discussion paragraph to incorporate the clinical significance beginning on line 258:

*“Although complete GDMT at discharge did not reach statistical significance—likely reflecting existing prescribing habits and limited integration time—the observed positive trend remains clinically important given GDMT’s known survival benefits. Notably, the significant improvement in ACEI/ARB/ARNI use is particularly impactful due to their well-established benefits on survival and hospitalization rates.”*

Minor comment:

**1) Page 6, Line 123: I would suggest that authors specify which guidelines they refer to at this point.**

We have added the citation to the 2022 AHA/ACC/HFSA guidelines.

**Reviewer #5: I would like to thank the authors for their contribution and the manuscript. The study indeed is significant and it’s encountered commonly in clinical practice due to failure to achieve GDMT in heart failure.**

Thank you for this comment.

**Reviewer #6: References need to be rewritten according to the journal's format.**
**Some abbreviations need clarification.**
**Images and tables are missing.**
**Please rewrite them with images and tables to make the idea clear to the reader.**

Tables have been added to the manuscript. All abbreviations have been carefully reviewed for accuracy. Images are available and have been uploaded separately.
